# Intractable parastomal bleeding in a portal hypertensive patient managed by direct sclerotherapy: a case report 

**Published:** 2020

**Authors:** Niloofar Ayoobi Yazdi, Najmeh Aletaha, Mohammad-Mehdi Mehrabinejad, Ali Zare Dehnavi, Hadi Rokni Yazdi

**Affiliations:** 1 *Department of Radiology, Advanced Diagnostic and Interventional Radiology Research Center(ADIR), Tehran University of Medical Sciences, Tehran, Iran*; 2 *Department of Gastroenterology, Tehran University of Medical Sciences, Tehran, Iran*; 3 *Students Scientific Research Center, Tehran University of Medical Sciences, Tehran, Iran *

**Keywords:** Sclerotherapy, Gastrointestinal hemorrhage, Transjugular intrahepatic portosystemic shunt

## Abstract

Patients with a stoma have 5% chance of developing parastomal varices, which tend to repetitive massive and life-threatening hemorrhages. Treatment of choice in parastomal varices have not been established, while Transjugular Intrahepatic Portosystemic Shunt (TIPS) has been revealed as the most successful measure. We report a hemodynamically unstable patient with a history of Ulcerative Colitis (UC) and Primary Sclerosing Cholangitis (PSC) with colostomy, because of colon cancer who presented with massive parastomal bleeding. Non-operative treatments and TIPS failed to control the symptoms. Color Doppler ultrasound showed a hepato-fugal flow. The direct antegrade technique, using Sodium Tetradecyl Sulfate (STS 1%) and glue-Lipiodol, was applied under ultrasonography guidance, and complete stoppage of bleeding was achieved. No immediate or late complication or follow-up recurrence were noted after 8 months. In case of hepatofugal flow, direct percutaneous mesenteric parastomal venous access and sclerotherapy is a rapid and relatively safe procedure for parastomal variceal bleeding.

## Introduction

 Stoma (colostomy or ileostomy) develops due to various pathologies, with Ulcerative Colitis (UC) being the most prevalent cause (1). It has associated with several problems, including Parastomal Varices, Portal venous obstruction, and consequently portal hypertension, induced by liver cirrhosis and Primary Sclerosing Cholangitis (PSC), as the most prevalent underlying diseases. They also culminate in parastomal varices at the portosystemic junction (2). Patients with a stoma have a 5% chance of developing parastomal varices, especially in men and if they are about 50 years old (3). There are limited specific signs and symptoms, such as raspberry appearance and visible submucosal veins, but none of them are pathognomonic. Although they are painless, they could eventuate in severe repetitive bleeding, and consequently up to 4% mortality. The most popular radiological diagnostic modalities are venous phase mesenteric angiography as well as portal venography (3). Likewise, Doppler ultrasound, triphasic CT scan or CT venography, and CT angiography are known as noninvasive and accurate modalities (4,5). Magnetic resonance imaging (MRI) can also be used to evaluate venous drainage especially in case of contraindication of iodinated contrast agent administration (6).

Over the past decades, various methods have been implemented to reduce the parastomal hemorrhage volume and frequency. Compression dressing, ligation, and beta-adrenergic blockers are considered as the first-line management measures, with almost 75% of them being managed by local measures. Then, local surgical treatments such as revision and relocation of stoma are offered to the patients when these attempts fail. Portosystemic shunt, surgical or transjugular, are used extensively in order to control the long-term variceal hemorrhage. Transjugular Intrahepatic Portosystemic Shunt (TIPS) has been recognized as the most successful option in the bleeding frequency reduction with the re-bleeding rate of 20%. Embolization and sclerotherapy are the additional options to tackle this problem, where sclerotherapy can result in retraction, ulceration, and stricturing of the stoma (3). Liver transplant is recommended for the patients with no less invasive alternative (7).

We have studied a case of UC & PSC with colostomy, because of colon cancer who presented with massive stomal bleeding, which was controlled finally with direct parastomal sclerotherapy & glue injection, without any complication. 

## Case Report

A 52-year-old man was admitted to Imam Khomeini medical university hospital for recurrent bleeding from the treatment site of colostomy. The patient signed consent form and was assured that his individual data would remain confidential to the research team. The patient had a history of UC, PSC, cholecystectomy, and underwent left hemicolectomy with a colostomy because of colon cancer in August 2018. Surgery was followed by chemotherapy.

Four months after surgery, bleeding started from the colostomy site, and it was exacerbated progressively. Hemoglobin value reached as low as 2 mg/dl in the last bleeding episode, due to the frequent blood losses, and the patient required several hospitalizations. The patient’s heart was arrested in the last bleeding episode and was revived via resuscitation and blood transfusions.

 Local compression, ligation, and cauterization were also used as first-line management in order to control bleeding in continuing hospitalizations, but all failed. Consequently, TIPS was planned for the patient in April 2019, which was not successful. The patient was medically in poor condition for revision surgery, and was not a candidate either for a liver transplant. 

**Figure 1:**
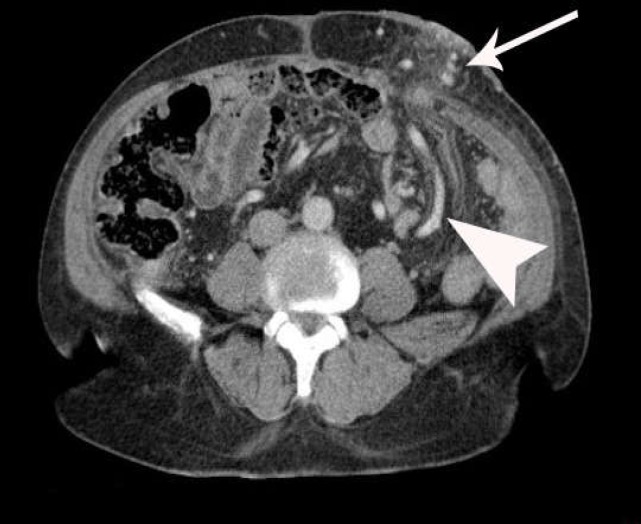
52 YO male with UC, PSC& colostomy due to colon cancer who presented with massive stomal bleeding. Arterio-portal phase CT SCAN shows parastomal varices (arrow) and enlarged mesenteric afferent tributary (arrowhead)

Venous phase CT scan indicated parastomal varices with an afferent vein from the inferior mesenteric vein tributaries, where efferent veins to multiple subcutaneous epigastric veins finally drained to the left iliofemoral junction (Figure1).

**Figure 2:**
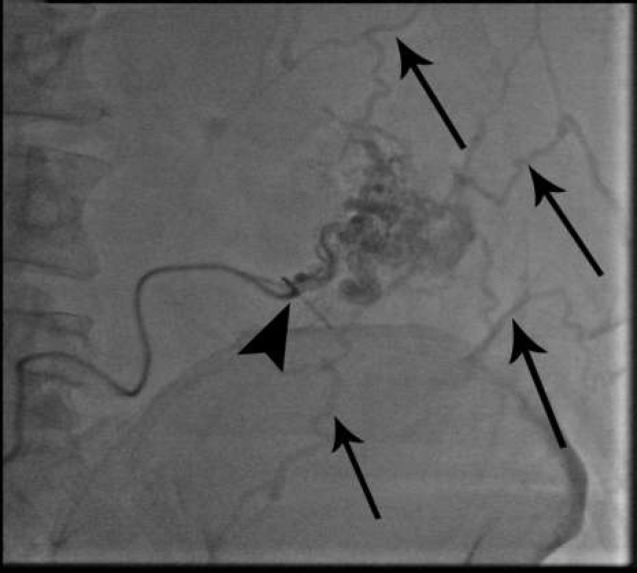
Ultrasound guide parastomal varicose vein puncture and contrast injection in inferomedial part of colostomy (mesenteric part) (arrowhead) and multiple afferent tiny subcutaneous systemic veins (arrows)

As a final point, sclerotherapy was prescribed for the patient in May 2019. Color Doppler ultrasound indicated a hepato-fugal flow; a 22-gauge scalp vein was inserted under the ultrasonography guidance inferomedial part of the colostomy to the varices on the mesenteric side; in contrast, injected varices were presented to drain into multiple small subcutaneous veins, and eventually drained to the left iliofemoral junction (Figure 2). The direct pressure on LLQ area was performed in order to avoid sclerosant agent systemic entrance. Obliteration of parastomal varices was applied using Sodium Tetradecyl Sulfate (STS 1%) via a routine two-syringe foam formation method. A negative contrast (air) was observed during the slow injection, which initially went into the afferent subcutaneous veins and then refluxed to the inferior mesenteric tributary vein. Next, we stopped injection (Figure 3A), and after that we injected 2CC of 70% glue-Lipiodol mixture (Figure 3B). 

**Figure 3A:**
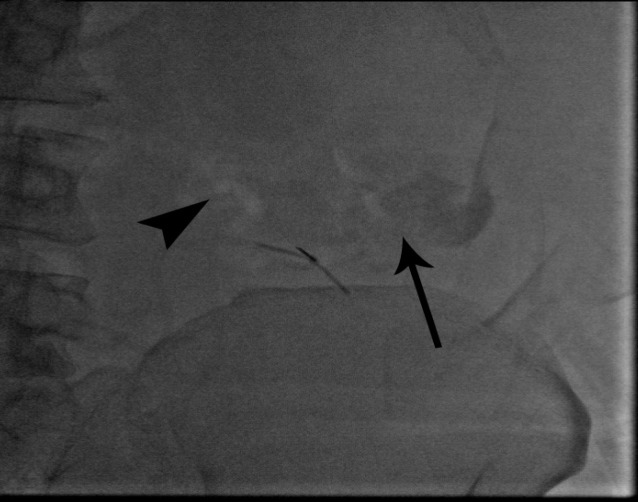
Foam sclerotherapy of parastomal varices with slow injection and using negative air contrast to follow the foam, the afferent systemic veins were firs visualized (arrow). Due to LLQ manual compression and efferent obstruction, reflux to afferent mesenteric side is noted (arrowhead)

**Figure 3B:**
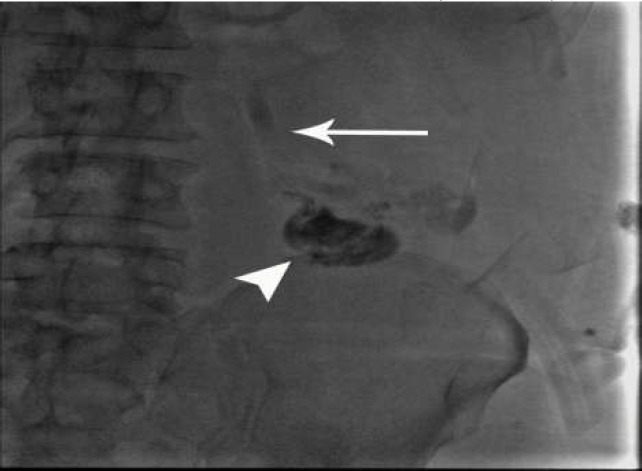
after sclerotherapy, contrast injected, some residual varicose veins noted, without obvious efferent veins and stagnation in IMV (arrow); then about 2 cc, 70%glue (arrowhead) was injected to prevent possible recurrence

The burning sensation at the injection site faded after 1 hour, and no immediate complications were observed following this procedure, with complete stoppage of bleeding accomplished. The patient with stable vital signs and symptoms relief was discharged from the hospital. CT scans after one week for ruling out possible complications indicated complete obliteration of varicose and large mesenteric tributary veins, with no thrombosis in other mesenteric, portal and systemic veins observed. No complication or follow-up recurrence were noted after 8 months. 

## Discussion

There are ample evidence indicating that varices develop at the portosystemic junctions, where high-pressure portal veins and low-pressure systemic veins are in connection, such as esophagus, rectum, periumbilical, and parastomal (8). Parastomal varices tend to be repetitive and life-threatening massive hemorrhages, which could require multiple blood transfusions (9). However, it is believed that parastomal variceal bleeding as a safety valve reduces esophageal variceal bleeding (10).

Gold standard methods of parastomal varices diagnosis and treatment have not been established, and various studies were carried out for introducing the best options (3). Management of parastomal varices requires a multidisciplinary approach including endoscopists, gastroenterologists, interventional radiologists, and surgeons (11).

The most popular options that have been cited include non-operative treatments (compression, ligation, sclerotherapy, propranolol, etc.), local operative treatments, embolization, surgical or transjugular intrahepatic portosystemic shunt, and liver transplant. Amongst all of these options, ligation and sclerotherapy are the least invasive procedures, while a liver transplant is provided to patients with no substitute (3). Most research has recommended TIPS as the most effective measure to reduce portal hypertension and re-bleeding risk accordingly (12). Approximately three-quarters of patients benefit from local measures as primary management, despite various advanced methods (3). Endoscopic therapies including endoscopic ligation, endoscopic sclerotherapy (ES), and endoscopic tissue adhesive (ETA), which are frequently used in esophageal varices, are also considered to be effective in such cases (13). ES and ETA lead to vessel thrombosis and hemostasis through tissue irritation or injury via injecting a sclerosant or tissue adhesive (13). On the other hand, complications of ES and ETA are almost similar and include transient dysphagia (70%), esophageal ulceration (60%), chest discomfort (65%), and low-grade fever (6–10%) (14). The risk of re-bleeding following these measures has also been reported as high as 20%-25% (15).

In this investigated case, bleeding failed to respond to the first-line management, including compression dressing and ligation. The patient underwent multiple packed cells transfusion, and TIPS also failed. Thus, sclerotherapy was provided to the patient, and complete stoppage of parastomal hemorrhage was achieved. Sclerotherapy in parastomal varices as a minimally invasive procedure has been employed since 1986 (9,10). Although sclerotherapy has proved to be an efficient measure for complete stoppage of bleeding (17), some undesirable damages may occur to stoma, including retraction, ulceration, and stricturing (18). 

Transvenous obliteration or sclerosis of parastomal varices includes the three following approaches: Balloon-occluded Retrograde Transvenous Obliteration (BRTO), Balloon-occluded Antegrade Transvenous Obliteration (BATO), and direct antegrade technique. BRTO is a systemic venous approach, which requires at least type-2 ectopic varices, and is considered as a time-consuming and grueling procedure. BATO is a portal venous approach, which is performed as Percutaneous Transhepatic (PTO) or trans-TIPS. The direct antegrade technique is the least invasive and a very quick procedure with the same efficacy in comparison with the other techniques. When the flow in the mesenteric venous feeder is detected as hepatofugal by color Doppler ultrasound, and toward the stoma, the direct antegrade technique can be performed. However, the hepatopedal or fluctuant flow requires a BRTO or PTO approach. We observed a hepatofugal flow in the mesenteric feeder, with direct antegrade technique applied. Performing this method in type-2 and 3 ectopic varices with systemic outflow requires ultrasound transducer compression in order to avoid sclerosant agent systemic entrance with no requirement for the balloon occlusion. This technique is recognized as the least invasive one as it does not require transhepatic or trans-TIPS access (19). 

We did not use a balloon occlusion, because of many tiny efferent systemic veins, and used focal compression of superficial veins as well as STS foam negative air contrast under fluoroscopy. Finally, we also injected a small amount of glue at the end of the procedure in order to reduce the recurrence rate.

In case of hepatofugal flow, direct percutaneous mesenteric parastomal venous access and sclerotherapy is a rapid and relatively safe procedure for parastomal variceal bleeding in unstable patients and in emergency situations.

## References

[1] Uchino M, Ikeuchi H, Bando T, Chohno T, Sasaki H, Horio Y (2017). Is An Ostomy Rod Useful for Bridging the Retraction During the Creation of a Loop Ileostomy? A Randomized Control Trial. World J Surg.

[2] Saad WE, Saad NE, Koizumi J (2013). Stomal varices: management with decompression tips and transvenous obliteration or sclerosis. Tech Vasc Interv Radiol..

[3] Pennick MO, Artioukh DY (2019). Management of parastomal varices: who re-bleeds and who does not? A systematic review of the literature. Tech Coloproctol.

[4] Goldstein WZ, Edoga J, Crystal R (2019). Management of colostomal hemorrhage resulting from portal hypertension. Dis Colon Rectum.

[5] Jae WC, Chang HL, Kyeong AK, Cheol MP, Jin YK (2006). Ectopic varices in colonic stoma: MDCT findings. Korean J Radiol.

[6] Minami S, Okada K, Matsuo M, Kamohara Y, Sakamoto I, Kanematsu T (2007). Treatment of bleeding stomal varices by balloon-occluded retrograde transvenous obliteration. J Gastroenterol..

[7] Hedaya MS, El Moghazy WM, Uemoto S (2019). Living-related liver transplantation in patients with variceal bleeding: outcome and prognostic factors. Hepatobiliary Pancreat Dis Int.

[8] Johnson PA, Laurin J (2019). Transjugular portosystemic shunt for treatment of bleeding stomal varices. Dig Dis Sci.

[9] Strauss C, Sivakkolunthu M, Ayantunde AA (2019). Recurrent and troublesome variceal bleeding from parastomal caput medusae. Korean J Gastroenterol.

[10] Thompson J (2019). Caput medusae: peristomal varices. J ET Nurs.

[11] Garcia-Tsao G, Abraldes JG, Berzigotti A, Bosch J (2017). Portal hypertensive bleeding in cirrhosis: Risk stratification, diagnosis, and management: 2016 practice guidance by the American Association for the study of liver diseases. Hepatology.

[12] Kochar N, Tripathi D, McAvoy NC, Ireland H, Redhead DN, Hayes PC (2019). Bleeding ectopic varices in cirrhosis: the role of transjugular intrahepatic portosystemic stent shunts. Aliment Pharmacol Ther.

[13] Al-Khazraji A, Curry MP (2019). The current knowledge about the therapeutic use of endoscopic sclerotherapy and endoscopic tissue adhesives in variceal bleeding. Exp Rev Gastroenterol Hepatol.

[14] Rajoriya N, Tripathi D (2014). Historical overview and review of current day treatment in the management of acute variceal haemorrhage. World J Gastroenterol.

[15] Laine L, Deborah Cook MD (1995). Endoscopic ligation compared with sclerotherapyfor treatment of esophageal variceal bleeding: A meta-analysis. Ann Int Med.

[16] Morgan TR, Feldshon SD, Tripp MR (2019). Recurrent stomal variceal bleeding Successful treatment using injection sclerotherapy. Dis Colon Rectum.

[17] Hesterberg R, Stahlknecht CD, Röher HD (2019). Sclerotherapy for massive enterostomy bleeding resulting from portal hypertension. Dis Colon Rectum.

[18] Spier BJ, Fayyad AA, Lucey MR, Johnson EA, Wojtowycz M, Rikkers L (2008). Bleeding stomal varices: case series and systematic review of the literature. Clin Gastroenterol Hepatol.

[19] Saad WE, Saad NE, Koizumi J (2019). Stomal varices: management with decompression TIPS and transvenous obliteration or sclerosis. Tech Vasc Interv Radiol.

